# Evaluation of interprofessional education: lessons learned through the development and implementation of an interprofessional seminar on team communication for undergraduate health care students in Heidelberg – a project report

**DOI:** 10.3205/zma001021

**Published:** 2016-04-29

**Authors:** Sarah Berger, Cornelia Mahler, Katja Krug, Joachim Szecsenyi, Jobst-Hendrik Schultz

**Affiliations:** 1 Universitätsklinikum Heidelberg, Abteilung Allgemeinmedizin und Versorgungsforschung, Heidelberg, Germany; 2 Universitätsklinikum Heidelberg, Klinik für Allgemeine Innere Medizin und Psychosomatik,Heidelberg, Germany

**Keywords:** communication, competency-based education, interdisciplinary health team, interprofessional relations, professional education

## Abstract

**Introduction: **This project report describes the development, “piloting” and evaluation of an interprofessional seminar on team communication bringing together medical students and Interprofessional Health Care B.Sc. students at the Medical Faculty of Heidelberg University, Germany.

**Project Description: **A five-member interprofessional team collaborated together on this project. Kolb’s *experiential learning* concept formed the theoretical foundation for the seminar, which explored three interprofessional competency areas: team work, communication and values/ethics. Evaluation for the purposes of quality assurance and future curricula development was conducted using two quantitative measures:

1. descriptive analysis of a standardized course evaluation tool (*EvaSys*)
2. ANOVA analysis of the German translation of the University of the West of England Interprofessional Questionnaire (UWE-IP-D).

**Results: **The key finding from the standardized course evaluation was that the interprofessional seminars were rated more positively [M=2.11 (1 most positive and 5 most negative), SD=1, n=27] than the monoprofessional seminars [M=2.55, SD=0.98, n=90]. The key finding from the UWE-IP-D survey, comparing pre and post scores of the interprofessional (IP) (n=40) and monoprofessional (MP) groups (n=34), was that significant positive changes in mean scores for both groups towards communication, teamwork and interprofessional learning occurred.

**Conclusions: **Lessons learnt included: a) recognising the benefit of being pragmatic when introducing interprofessional education initiatives, which enabled various logistical and attitudinal barriers to be overcome; b) quantitative evaluation of learning outcomes alone could not explain positive responses or potential influences of interprofessional aspects, which highlighted the need for a mixed methods approach, including qualitative methods, to enrich judgment formation on interprofessional educational outcomes.

## Zusammenfassung

**Einleitung: **Dieser Projektbericht beschreibt die Entwicklung, Pilotierung und Evaluation eines interprofessionellen Seminars zur Team-Kommunikation, das Studierende der Humanmedizin und Studierende des Studiengangs Interprofessionelle Gesundheitsversorgung B.Sc. an der Medizinische Fakultät der Universität Heidelberg zusammengebracht hat.

**Projektbeschreibung: **Ein fünfköpfiges interprofessionelles Team arbeitete zusammen an diesem Projekt. Kolbs Theorie des *erfahrungsorientierten Lernens* bildete die theoretische Grundlage für das Seminar, in dem drei interprofessionelle Kompetenzdomänen vermittelt wurden: Teamarbeit, Kommunikation und Werte/Ethik. Die Evaluation zum Zwecke der Qualitätssicherung und der zukünftigen curricularen Entwicklung wurde mit zwei quantitativen Instrumenten durchgeführt:

1. deskriptive Analyse eines standardisierten Kurs-Bewertungsinstruments *(EvaSys) *
2. Varianzanalyse (ANOVA) der deutschen Version des Fragebogens „University of the West of England Interprofessional Questionnaire“ (UWE-IP-D).

**Ergebnisse: **Das Hauptergebnis der standardisierten Kursevaluation war, dass die interprofessionellen Seminare positiver bewertet wurden [MW=2,11 (Bereich: 1 sehr positiv bis 5 sehr negativ), SD=1, n=27] als die monoprofessionellen Seminare [MW=2,55, SD=0,98, n=90]. Der Vergleich der Vorher-/Nachher-Ergebnisse in der UWE-IP-D Befragung der interprofessionellen (IP) (n=40) und monoprofessionellen (MP) Gruppen (n=34) zeigte signifikante positive Veränderungen der Mittelwerte für beide Gruppen hinsichtlich Kommunikation, Teamarbeit und interprofessionellem Lernen.

**Schlussfolgerungen: **Erkenntnisgewinne aus dem Projekt: a) Nutzen einer pragmatischen Vorgehensweise bei der Einführung interprofessioneller Ausbildungssequenzen, die es ermöglichte, dass verschiedene Barrieren bzgl. Logistik sowie Einstellung/Haltung überwunden werden konnten; b) eine quantitative Evaluation allein konnte die positiven Rückmeldungen des interprofessionellen Settings nicht erfassen. Dies erfordert einen Mixed-Methods-Ansatz inklusive qualitativer Methoden, um die Effekte interprofessioneller Ausbildung besser zu verstehen.

## Authors

Shared first authorship (Berger and Mahler)

## Introduction

In today’s complex and dynamic health care environments, and with increasing numbers of patients requiring long-term care for chronic illness, health professionals need to be able to collaborate effectively in interprofessional teams to ensure safe, quality patient outcomes [1], [2], [3]. To enhance collaborative practice for the benefit of patients, in recent years, key agencies and joint collaboratives from the United Kingdom (2006) [4], Canada (2010) [5], the United States (2011) [6] and Australia (2011) [7] have each produced comprehensive frameworks outlining core interprofessional capabilities and/or competencies, which among others include: interprofessional communication skills, teamwork abilities, understanding other team members’ professional roles and responsibilities, ethical practice and reflection [4], [5], [6], [7]. These are intended to build upon discipline-specific capabilities and competencies in the health professions. These frameworks are also indispensable resources for educators of the health professions that can be used to support the development of interprofessional education initiatives. 

The U.K. Centre for the Advancement of Interprofessional Education (CAIPE) proposes the following definition of interprofessional education: “interprofessional education occurs when two or more professions learn with, from and about each other to improve collaboration and the quality of care” [8]. Importantly, the World Health Organisation *Framework for Action on International Education & Collaborative Practice* [9] highlights the role that interprofessional education has to prepare today’s undergraduate health science students as a “collaborative practice-ready health workforce” not only with discipline-specific competencies, but with interprofessional capabilities and competencies that will be required for their future collaborative practice in interprofessional health care teams. There is significant impetus at present to integrate interprofessional education into undergraduate health science curricula. 

Although this is an emergent field in the education of the health professions, there is already a solid foundation of research and a growing base of evidence. To date, this has largely focused on defining a conceptual and theoretical basis for the interprofessional education movement [10], [11], [12], [13] as well as developing sustainable models for the implementation of interprofessional education initiatives [14], [15], [16], [17], [18], [19], [20]. There has also been work done to identify suitable approaches for evaluation of interprofessional education [21], [22], [23], [24]. Where teaching strategies for interprofessional education in the undergraduate academic setting have been reported, this has included, for example, small-group discussions, problem-based learning or case analysis and simulation exercises [25], [26], [27], [28]. Nevertheless, to date, few studies have reported using a formal theoretical or conceptual framework as a foundation for the development of their interprofessional education project [23]. There is a need for further research in the field of interprofessional education so that reliable evidence continues to be made available to support educators in planning learning and teaching processes and assessment, to assist organizational decision-makers in addressing the logistical and systems-level issues to do with interprofessional collaboration, and importantly, to provide policy-makers with evidence about the long-term impacts of interprofessional education and collaboration on clinical care and patient outcomes.

Keeping pace with international trends in the interprofessional education and collaboration movement, in 2010, the Medical Faculty at Heidelberg University established a Bachelor of Science - Interprofessional Health Care, which has a competency-based curriculum designed for health care students [14], [15]. Students accepted into this limited entry programme are able to complete university studies in parallel to vocational training in a health profession, which allows them to achieve two qualifications (one academic and one professional) in four and a half years. The structure of this dual qualification programme has arisen due to current laws regulating the education of many health professional groups (*Gesundheitsberufe)* in Germany [14]. This dual qualification programme is, therefore, provided through a formal partnership between the Medical Faculty at Heidelberg University and the Academy for Health Professions, University Hospital Heidelberg. The Academy for Health Professions is a local vocational training provider for the following health professions: medical radiography (MTRA); medical laboratory science (MTLA); midwifery; general, geriatric and paediatric nursing; orthoptics; physiotherapy; and speech and language therapy.

Due to the fact that there are now both medical students and Bachelor of Science – Interprofessional Health Care students studying at undergraduate level, interprofessional education between these two groups can be offered at appropriate curricula interfaces to facilitate the development of interprofessional competencies in these health care students. This project report describes our first undergraduate interprofessional education project bringing together both medical students and bachelor students at the Medical Faculty at Heidelberg University through a mandatory seminar on team communication in the Winter Semester 2012/2013. 

## Project Description

This project report outlines the development, implementation and evaluation strategies for the interprofessional education seminar on team communication for medical students and bachelor students.

### Project objective

The overarching aim of this undergraduate interprofessional education project was to develop, “pilot” and evaluate the seminar on team communication. 

#### Interprofessional Project Team

A five-member interprofessional project team was brought together with representatives from the disciplines of medicine, nursing, sociology and psychotherapy. Most team members had additional qualifications in learning and teaching. The co-leaders – medicine (J-HS) and nursing (CM) – each held a pivotal role at programme level in the Medical Faculty (J-HS as Head of Medical Education, Department of Internal Medicine and CM as Coordinator – Bachelor of Science programme). The co-leaders shared responsibility for strategic planning and direction of the project. They were supported by three project team members who carried out the organizational and administrative steps of the project. 

#### Design and Development

Working in close collaboration with each other, the project team carried out the following steps in the design and development of the team communication seminar: establishment of a conceptual framework; design and development of teaching resources including learning objectives and expected outcomes; scheduling the seminar into the two curricula over one winter semester; recruitment of teaching staff; delivery of information sessions for teaching staff; assistance with preparation/set-up for the team communication seminars and overseeing the evaluation processes. The project team met regularly over approximately six months during the design and development stage, prior to the implementation of the team communication seminar in the Winter Semester 2012/2013.

Consultation with local stakeholders was also undertaken during this stage. For example, feedback on learning goals and instructional content was sought from knowledge experts. Also, feedback from the student perspective was sought by holding a one-off partial practice run of the seminar during the semester break with a small group of senior student tutors, before the team communication seminar was rolled out formally the following winter semester.

#### Conceptual Foundation

The team communication seminar was grounded in the principles of adult learning in education, and Kolb’s *experiential learning* concept formed the theoretical foundation for the seminar design [29]. 

#### Learning Context

In order to maximize gains from the experiential learning process, the small-group format was chosen as an appropriate type of learning context. This was to allow plenty of opportunity for direct interaction and exchange among the health care students. 

#### Teaching Strategies

Teaching strategies selected for the team communication seminar included the use of structured group exercises and role plays to enhance the experiential learning process. At the end of each planned activity, time was set aside for formal reflection and discussion. Also, to integrate an additional feedback source from peers and, thereby, enhance the reflection process, each activity had a few students rotating through an observation role rather than an active role. Their observation role was supported with short handouts outlining potential focus points; in addition, assistance from teaching staff was available when questions arose.

#### Instructional Content

For pragmatic reasons *(see Discussion-Process Learnings)*, the team communication seminar was designed to be delivered as a single 3½ hour session. During this time period, three primary themes related to three interprofessional competencies were explored by the students:

- Teamwork: based on a structured group exercise and involved students working together to complete a complex, non-clinical task i.e. to build up a three-dimensional puzzle.
- Communication: based on a “first aid” role play scenario in a community setting, which was video-recorded and replayed during the reflection stage. Students were supported to analyze and evaluate their interpersonal interactions and communication strategies.
- Values/ethics: based on an “Ethics Committee” role play and involved students discussing three complex clinical cases and exploring ethical issues related to organ transplantation.

Through this series of “experiences”, the health care students were introduced in an active way to three key interprofessional competency areas important for effective collaboration in health care teams. The Interprofessional Capability of Reflection was formally facilitated by the teaching staff at the end of each activity based on discussion of the students’ experiences. These reflection processes also integrated peer feedback from the student observers. Teaching staff introduced theoretical content related to each topic during the discussions at each of the three reflection stages. They also facilitated group dialogue on the experience of the interprofessional learning context as an additional phenomenon impacting on learning processes.

#### Scheduling

The team communication seminar was integrated into communication modules in the core curricula for the undergraduate medicine and bachelor programmes. Attendance was made mandatory for both groups. The bachelor programme has limited entry for up to 25 students per year commencing each winter semester. In contrast, there are over 160 medical students enrolled each semester. In order to accommodate these student numbers, the single 3½ hour team communication seminar was repeated 18 times during the winter semester with small groups of approximately 10-12 students. However, due to the large difference in numbers between the two student bodies, only four seminars could be made up as interprofessional groups. With the much larger number of medical students, a further 14 seminars were run as monoprofessional seminars for only medical students, although the interprofessional competency-based instructional content remained the same. 

#### Group allocation 

Medical students were assigned to small groups by a faculty secretary at the beginning of the semester and timetabled for different classes in their medical studies as part of their HeiCuMed curriculum at the Medical Faculty. This allocation process included the team communication seminars. Four small groups of medical students just happened to have been scheduled in the timeslots where the seminar was run with interprofessional groups. The 25 bachelor students were allocated to small groups on the scheduled dates according to health profession, so that a mixture of three or more health professions per small group was achieved thereby creating the desired interprofessional learning context.

#### Level of Learners

Bachelor students were in the third semester of study and medical students were in the sixth/seventh semester of study. 

#### Interprofessional Teaching Teams

Highly experienced teachers co-facilitated the four team communication seminars with interprofessional groups and worked in interprofessional teaching tandems (medical and nursing). Of the further 14 monoprofessional seminars, only four were co-facilitated by interprofessional teaching tandems, these served as a monoprofessional “control group” for the evaluation. Once again, due to the much larger number of medical teaching staff in the faculty, 10 monoprofessional groups were co-facilitated by only medical teaching staff.

#### Evaluation

Evaluation of the team communication seminar was carried out using two quantitative measures to systematically appraise learning outcomes according to Level 1 and Level 2 of Kirkpatrick’s outcomes typology [30]:

a) Level 1 evaluation was to appraise the *“initial reactions”* of what students thought and felt about the seminar. This was collected using standardized paper-based Medical Faculty course evaluation forms *(EvaSys).* These had 31 items (29 items had a five-point rating scale, there was also 2 items for free text comments). Of the 29 items, 6 items related to learning success, 7 items related to knowledge gains, 10 items related to general seminar features/organisation and 6 items related to teacher evaluation. These forms were administered directly after the team communication seminar and descriptive analysis with t-test was carried out; 

b) Level 2 evaluation was to appraise potential *“changes in attitude”* in response to the seminar. This was carried out using a validated survey instrument i.e. the German translation of the University of the West of England Interprofessional Questionnaire (UWE-IP-D). The survey was administered in paper-based form. Pre and post measures were made comparing attitudes to communication, teamwork and interprofessional learning with the interprofessional group (IP) and the monoprofessional “control group” (MP). Two scales were used: Communication and Teamwork [9 items; sum score range: 9 most positive to 36 most negative] and Interprofessional Learning [9 items; sum score range: 9 most positive to 45 most negative]. Mean scores for the IP group and the MP group were compared using ANOVA analysis.

The primary purpose of this two-level evaluation approach was for quality assurance, i.e. to evaluate the provided education by measuring immediate responses of students to the interprofessional seminar. Evaluation results were also used for subsequent curricula planning of further interprofessional education within the Medical Faculty.

Evaluation of Level 3 and Level 4 of Kirkpatrick’s outcomes typology was outside the scope and timeframe of this project. Level 3 *“behaviours”* seeks to measure longer-term impacts of education or training (such as transfer of knowledge into practice/on the job), which normally occurs three to six months afterwards. Level 4 *“results”* seeks to measure long-term outcomes of education or training (e.g. for health care education this would be at the level of patient care outcome measures) [30]. 

## Results

This section provides an overview of key evaluation results from the interprofessional education project. 

### Student attendees

A total of 165 students attended the team communication seminar in the Winter Semester 2012/2013. The seminar was delivered a total of 18 times to small groups of 10-12 students. Fourteen small groups were monoprofessional because of the much larger student body studying medicine, with a total of 125 students. Four small groups were interprofessional, with a total of 40 students (20 bachelor students and 20 medical students).

#### Standard Evaluation tool – EvaSys

The completion of the evaluation forms was voluntary and anonymous: 117 out of 165 potential response forms were returned. The standardized course evaluation form was used to evaluate *initial reactions* to what students thought and felt about the team communication seminar. The key finding was that the interprofessional seminars were evaluated slightly more positively [M=2.11 (1 most positive and 5 most negative), SD=1, n=27] than in the monoprofessional seminars [M=2.55, SD=0.98, n=90]. Interestingly, the mean scores for the interprofessional groups (IP) on self-reported knowledge gains in the area of “group dynamics” (i.e. role awareness and ability for self-reflection) were also higher than the monoprofessional groups (MP) [M(IP)=3.96; M(MP)=3.24; p<0.01]. 

#### Interprofessional Survey Instrument – UWE-IP-D

**Figure 1:**
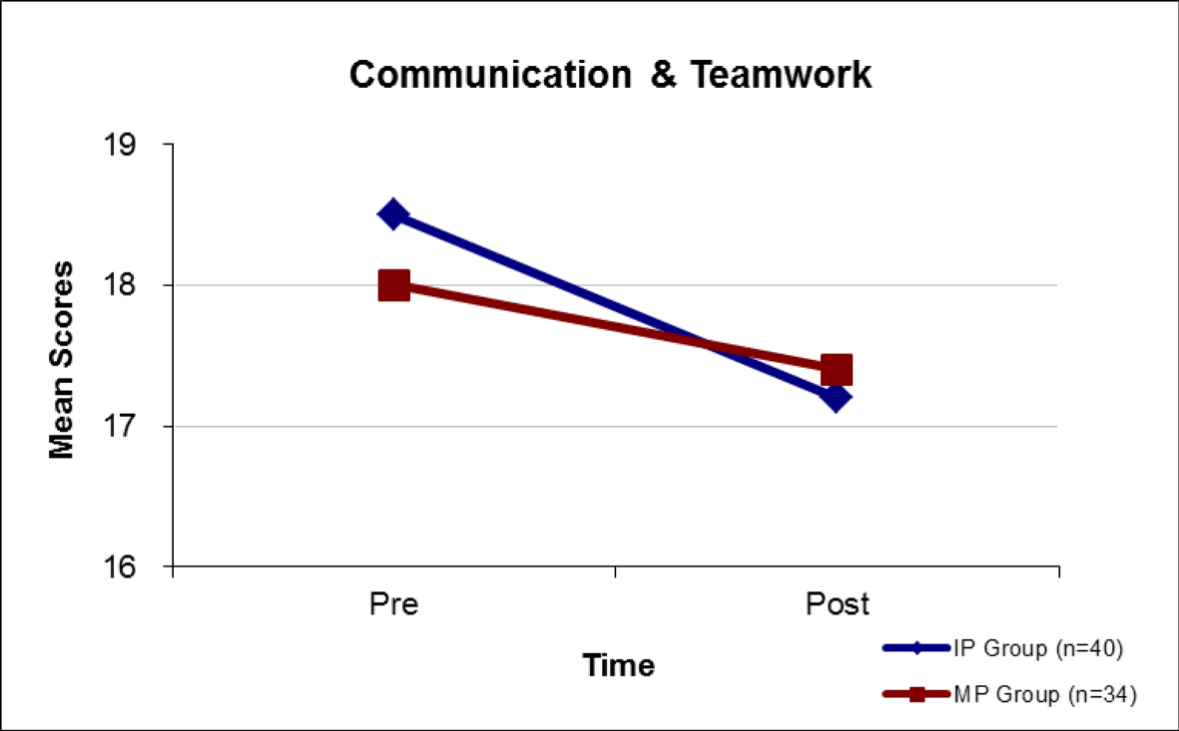
Attitudes to “Communication & Teamwork” (MP Group versus IP Group). Communication & Teamwork Scale: 9 items; 5-point Likert scale; Sum score range: 9 most positive to 36 most negative

The completion of the survey was voluntary and pseudonymous (to allow pre/post response matching): 40 out of 40 potential ‘pre&post’ responses were returned from the interprofessional group; 34 out of 36 potential ‘pre&post’ responses were returned from the monoprofessional control group. The UWE-IP-D survey was used as an outcome measure to evaluate potential *changes in attitude* as a result of the seminar. Quantitative results of the UWE-IP-D survey from the interprofessional (IP) group (n=40) were compared with a monoprofessional (MP) “control group” (n=34). The key findings in the comparison of pre and post results showed significant positive changes in mean scores for both groups towards communication, teamwork and interprofessional learning, which suggested that the educational seminar had a positive impact: 

Communication and Teamwork Scale (see Figure 1 (Fig. 1)): 

baseline mean scores for both groups were similar (Pre: M(IP)=18.5, M(MP)=18.0; p=0.82) and afterwards both showed significant (p<0.01) positive changes (Post: M(IP)=17.2, M(MP)=17.4). [Sum score range: 9 most positive to 36 most negative].

**Figure 2:**
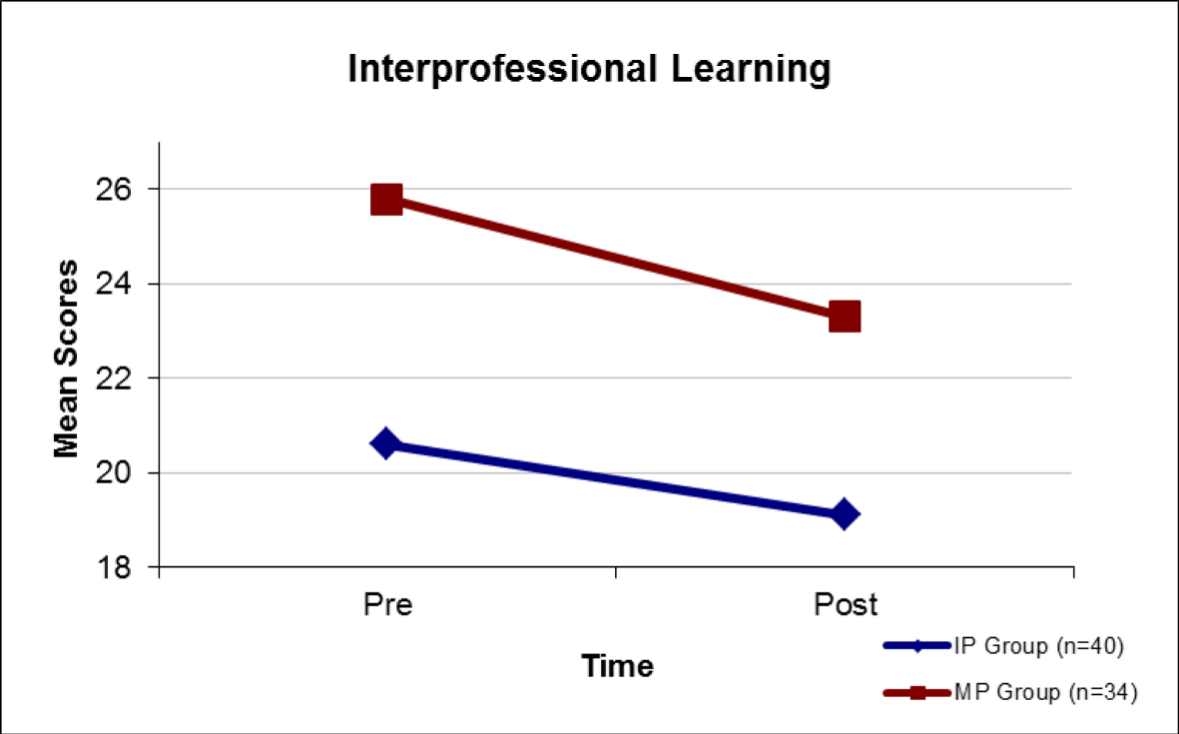
Attitudes to “Interprofessional Learning” (MP Group versus IP Group). Interprofessional Learning Scale: 9 items; 5-point Likert scale; Sum score range: 9 most positive to 45 most negative

Interprofessional Learning Scale (see Figure 2 (Fig. 2)): 

the IP group had a more positive baseline mean score (Pre: M(IP)=20.6, M(MP)=25.8; p<0.01). However, both groups showed significant (p < 0.01) positive changes (Post: M(IP)=19.1, M(MP)=23.3). [Sum score range: 9 most positive to 45 most negative].

However, when change in attitude between groups was compared, this was not significantly different, meaning potential influences of the interprofessional factor were not revealed using this quantitative measure. 

## Discussion

The overarching goal to develop and implement an interprofessional seminar on team communication was successfully achieved, and accompanying quantitative evaluation was generally positive. Nevertheless, as this was our first interprofessional education project not everything ran smoothly and in this section, we will discuss key lessons learned.

### Process Learnings

Our key process learning was that there was a need to be pragmatic in order to overcome potential logistical and attitudinal barriers that arose with the introduction of an interprofessional education seminar into undergraduate health science curricula. For example, timetabling the team communication seminar across already densely packed curricula in the undergraduate medicine and bachelor programmes was a significant logistical challenge. Ultimately, one 3½ hour timeslot in an existing communication module in the undergraduate medical curriculum was identified as an adaptable target. The interprofessional seminar on team communication took over this timeslot and could, therefore, be integrated into core curricula for both the undergraduate medicine and bachelor programmes. 

In addition, a further logistical challenge that arose was trying to coordinate information sessions for busy clinical staff from different departments, who would be teaching together in the team communication seminars in the Winter Semester 2012/2013. Difficulties with this meant that only one face-to-face information session for all 14 teaching staff (those for both the interprofessional and monoprofessional groups) could be scheduled just prior to the beginning of the winter semester. Additional information had to be provided on an individual basis (which was time consuming for the project team) and in electronic form by email. The minimal preparation provided for teaching staff was considered a limitation of this project.

Furthermore, there were concerns from some teaching staff about the potential challenge of teaching in an interprofessional classroom setting for those accustomed to teaching monoprofessional groups. This was addressed pragmatically i.e. highly experienced teachers (in monoprofessional settings) that had an expressed interest in being involved in the “pilot” interprofessional seminars facilitated our first seminars.

Finally, some attitudinal barriers were encountered at faculty level to the idea of introducing interprofessional education for both medical and bachelor students. The project team was willing to compromise and look for workable solutions. This resulted in the decision to start small with a “pilot” project. This was seen as an acceptable middle way for both enthusiasts and initial resisters to this curricula change for the introduction of interprofessional education. 

#### Lessons learned - Evaluation

The two quantitative measures to systematically appraise learning outcomes according to Level 1 and Level 2 of Kirkpatrick’s outcomes typology proved useful in that we could effectively demonstrate the immediate positive learning outcomes of our interprofessional seminar to curricula decision-makers within the Medical Faculty. The overarching goal of this project was successfully achieved; the interprofessional seminar on team communication has been embedded into the curricula for both medical students and bachelor students, and it now takes place on an annual basis each winter semester.

However, as a project group, we saw the results of the quantitative evaluation as a limited gain as we could not explain specifically why there were positive responses nor could we capture effectively potential influences of the interprofessional factor on these positive responses. It was only with the benefit of hindsight that we recognized how useful an accompanying qualitative evaluation might have been. 

Therefore, in the process of reflection and review at the end of the project, the interprofessional project team identified the need to adopt a mixed methods approach to evaluation design and recognized the value of collecting both quantitative and qualitative evaluation data for subsequent interprofessional education initiatives. A recent paper on evaluation of interprofessional education by Reeves, Boet, Zierler and Kitto (2015) supports this conclusion [31].

#### Further developments

New goals arising out of this interprofessional education project were: to build on initial experiences and seek curricula interfaces for additional interprofessional education seminars for medical students and bachelor students and to raise the profile of interprofessional education generally at the Medical Faculty. The project team has taken its pragmatic approach forward in the development of further interprofessional education seminars at our Medical Faculty and, since the Winter Semester of 2012/2013, it has found three further curricula interfaces for medical students and bachelor students. Interprofessional seminars on medical error communication, health care English and small business management are now also embedded in both programmes. A mixed methods approach to evaluation design collecting both quantitative and qualitative data has been implemented. A long-term strategic goal is to facilitate the development of interprofessional collaboration between health professions, through quality undergraduate interprofessional education, and thereby, to positively contribute to patient safety and quality health care outcomes in the future.

#### Afterword

The project to develop and implement an interprofessional seminar on team communication was formally completed at the end of the Winter Semester 2012/2013. Nevertheless, based on lessons learned regarding evaluation processes, the interprofessional project team was motivated to gather qualitative evaluation data driven by the unanswered questions as to why the seminar had generated positive responses and what potential influences the interprofessional learning context might have had. This led to the idea of conducting qualitative one-on-one interviews with the teaching staff about their experiences facilitating interprofessional education. Although, this took place outside the scope of the formal interprofessional education project reported here, we did subsequently interview the two doctors and the two nurses in the interprofessional teaching teams that co-facilitated the four interprofessional seminars and also co-facilitated the four monoprofessional seminars that formed our original “control group”. Results have been presented at a national conference [32]. In addition, a formal qualitative research study was designed and implemented with the cohort of medical and bachelor students that took part in the team communication seminar in the Winter Semester 2013/2014. Results of this study are currently being analyzed. These additional steps are allowing further in-depth analysis of this interprofessional seminar as part of on-going curricula development processes in interprofessional education at the Medical Faculty.

## Conclusions

Our key process learning was recognising the benefit of being pragmatic when introducing interprofessional education into undergraduate health science curricula. This enabled us to overcome logistical and attitudinal barriers that arose. In terms of evaluation, a key learning was that quantitative methods were effective in measuring immediate educational outcomes. However, this was a limited gain as we could not explain specifically *why* there were positive responses, nor could we capture effectively potential influences of the interprofessional factor on these positive responses. Based on these findings, subsequent evaluations of curricula changes that integrate interprofessional education have taken a mixed methods approach, including qualitative methods, to broaden and enrich judgment formation on educational outcomes.

## Acknowledgements

This project was supported by a grant from the Robert Bosch Stiftung, Germany. The authors thank Heike Lauber HPG and Daniela Suchy MA for their invaluable contributions to this first interprofessional education project. 

## Competing interests

The authors declare that they have no competing interests.
